# An approach to predict the height of fractured water-conducting zone of coal roof strata using random forest regression

**DOI:** 10.1038/s41598-018-29418-2

**Published:** 2018-07-20

**Authors:** Dekang Zhao, Qiang Wu

**Affiliations:** 1 College of Geoscience and Surveying Engineering, China University of Mining & Technology (Beijing), Beijing, 100083 China; 2 National Engineering Research Center of Coal Mine Water Hazard Controlling, Beijing, 100083 China

## Abstract

Water inrushes from coal-roof strata account for a great proportion of coal mine accidents, and the height of fractured water-conducting zone (FWCZ) is of significant importance for the safe production of coal mines. A novel and promising model for predicting the height of FWCZ was proposed based on random forest regression (RFR), which is a powerful intelligent machine learning algorithm. RFR has high prediction accuracy and is robust in dealing with the complicated and non-linear problems. Also, it can evaluate the importance of the variables. In this study, the proposed model was applied to Hongliu Coal Mine in Northwest China. 85 field measured samples were collected in total, with 60 samples (70%) used for training and 20 (30%) used for validation. For comparison, a support vector machine (SVM) model was also constructed for the prediction. The results show that the two models are in accordance with the field measured data, and RFR shows a better performance on good tolerance to outliers and noises and efficiently on high-dimensional data sets. It is demonstrated that RFR is more practicable and accurate to predict the height of FWCZ. The achievements will be helpful in preventing and controlling the water inrushes from coal-roof strata, and also can be extended to various engineering applications.

## Introduction

In mining activities, mine water has always been a great threat to the coal mine safety. According to statistics, more than 25 billion tons of coal resources are at the risk of water inrushes in China^1^. With increased mining depths in recent years, the hydrogeological conditions of mining become more and more complicated, and the water inrushes from coal-roof strata are increasingly serious^2,3^. During coal extraction, the strata overlying the coal seams move significantly downward due to the rock pressure, forming multiple fractures and fissures in the coal-roof strata. Once these fractures are interconnected and the impermeability of aquitards is destroyed, various kinds of water bodies from coal-roof strata, including surface water, goaf water and aquifer water, will flow into the mining area through the fractures, resulting in water-inrush accidents. The accidents may cause tremendous loss of life and property. Therefore, in order to effectively prevent water inrushes and ensure the safe production of the coal mines, it is essential to accurately predict the height of fractured water-conducting zone (FWCZ) of coal-roof strata.

Aiming at the prediction of the height of FWCZ, scholars proposed many methods, including empirical formula method, field measured method, theoretical calculation, numerical simulation and so on^4–12^. In the early 1980s, Liu^4^ proposed an empirical formula by the regression analysis of the limited field measured data collected from several large-scale coalmines in North China. But the formula only considers a few factors so that it is unable to precisely reflect the complicated development mechanism of the water-conducting fractures. For this problem, Hu^5^ summarized the nonlinear statistical relation between the FWCZ and multiple mining factors including mining height, hard-rock lithology ratio, working face length, mining depth and so on. Shi^6^ analyzed the movement characteristics of the overlying strata and the division theory of the “four zones” in overlying strata, then proposed theoretical formulas considering multiple mining factors. To ensure the mining safety of shallow coal seams under water-rich aquifers and determine the development of the fractured water-conducting zone, Liu^7^ built a numerical model to analyze the damage zone distribution in Flac3D model. Furthermore, the mathematical theories were gradually adopted in this field. For instance, Yang^8^ used the analytic hierarchy process and the fuzzy cluster analysis method to predict the height of FWCZ. Generally, the theoretical calculation model and the numerical simulation have the problems of over idealization and the difficult selection of the mechanical parameters. Meanwhile, the methods were also combined to determine the height of FWCZ. Based on the traditional empirical formula, the crack-measure system and the borehole television detecting system, Gao^9^ developed the theoretical calculation, quantitative analysis and detection of the FWCZ of coal-roof strata. At present, the most effective method is the field measurement by using borehole video camera system, water injection system and other direct monitoring equipment. However, these systems require large quantities of engineering with enormous expenses.

In recent years, with the rapid development of artificial intelligence technologies, the application of machine learning algorithms (MLAs), such as decision tree (DT), support vector machine (SVM), artificial neural network (ANN) and so on, to predicting the height of FWCZ has gradually been a trend^13–17^. For example, Sun^13^ proposed a synthetic calculation system that coupled genetic algorithm (GA) and support vector regression (SVR). This system reflected the relationship between the height of FWCZ and the mining factors effectively. Wu^14^ presented a radial basis function neural networks (RBFNN) model to predict the height of FWCZ for fully mechanized longwall mining with sublevel caving. However, these MLAs also have certain limitations in practical applications. For instance, numerous data pre-treatment is required in the DT model, and it tends to fall into local optimum; as for the ANN model, it has the shortcomings of over learning, slow convergence speed and local minimum value^2^.

Considering all the aforementioned problems, this paper proposed a predicting model of the height of FWCZ based on random forest regression (RFR), which is a non-parametric regression approach introduced by Breiman^18^ in 2001. RFR is a nonlinear modeling tool, coupling the main advantages of two major learning techniques: bagging and random feature selection^18^. It is suitable for the problems with unclear priori knowledge and incomplete data. Further, unlike simple DTs and neural networks (NNs), RFR runs efficiently on high-dimensional data sets. But if there are a lot of irrelevant variables, the DTs does not perform well. The objective of decision tree is to find the interaction between variables, and the weakness of the neural network is its inability to explain its reasoning process and reasoning basis^18^. Compared with the traditional intelligence algorithms, such as ANN and SVM, RFR has high prediction accuracy and good tolerance to outliers and noises^18,19^. Because of its superior performance, RFR has been widely applied to various fields such as biology, medicine, economics, management, remote sensing and other fields in recent years^19–25^. RBFNN has strong nonlinear fitting ability, it can map any complex nonlinear relation, and its learning rule is simple, which is easy to realize by computer. However, the theory and learning algorithm need to be further improved^26–29^. The group method of data handling (GMDH)-type neural network does not need the preset network structure, and the classification rules are expressed by some simple polynomials. However, the GMDH training algorithm can obtain good results only in the case that the noise and interference are distributed by Gaussian, otherwise, the training algorithm often overfits the network^30,31^. In the current study, RFR was used to predict the height of FWCZ. To verify the effectiveness of the generated RFR model, it was applied to Hongliu Coal Mine in Northwest China. Also, a SVM model was constructed for comparison. The results indicate that the RFR model has a better performance, and the prediction results are in good accordance with the field measured data observed using borehole video camera system (BVCS).

## Material and Methods

### Study area

**Figure 1:**
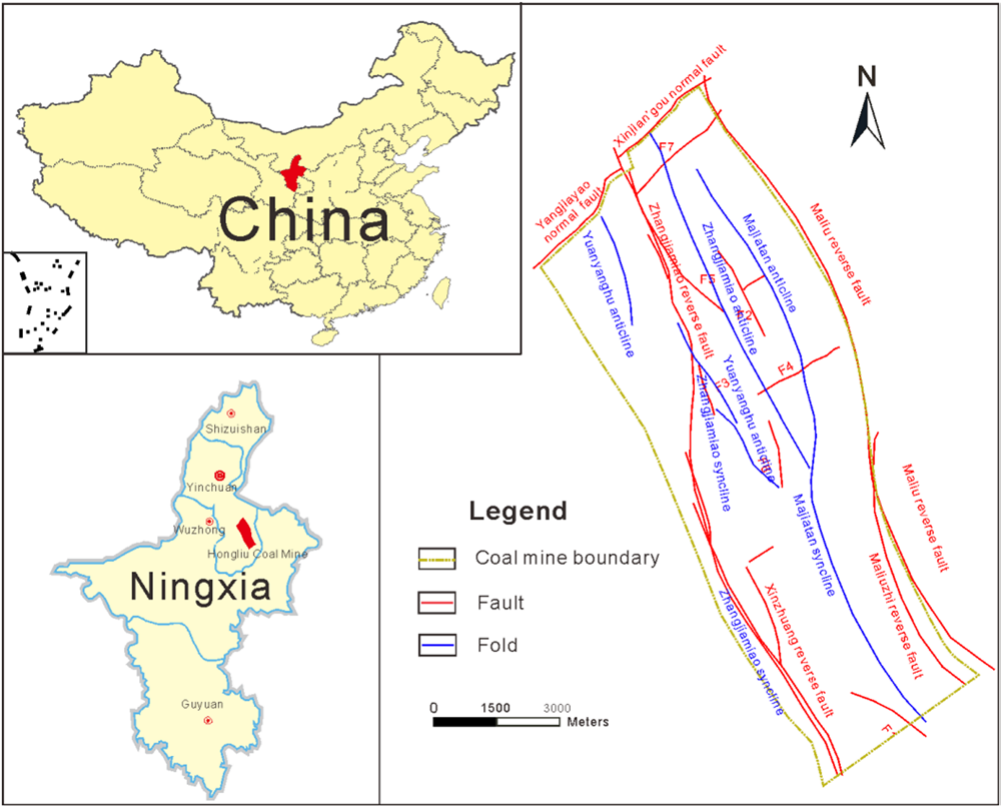
Location of the study area and geological structure map.

The Hongliu Coal Mine is situated in the middle east of the Ningxia Hui Autonomous Region in Northwest China, approximately 80 km northwest of Yinchuan City (Fig. 1). The mine is distributed in the NW-SE direction, and it has an area of 79.55 km^2^ with a length of 15 km and a width of 5.5 km. The general elevation is approximately 1400 m above sea level. Topographically, the mine is located in the west of Mu Us Desert, and the landform in the study area is classified as hilly terrain. It has a semiarid-desert continental-monsoon climate with a mean annual precipitation of 216.3 mm.

Most areas of the mine are covered by aeolian sand of Quaternary, except that sporadic bedrocks are exposed in certain local regions in the southwest of the mine field. According to drilling data, the main strata include: Shangtian Formation of Upper Triassic, Yan’an Formation of Medium Jurassic, An’ding Formation of Upper Jurassic, Qingshuiying Formation of Paleogene (Oligocene) and Quaternary.

Hydrogeologically, aquifers in the mine area can be divided into five groups according to the type of aquifer media and void: Quaternary loose alluvial pore aquifer group; Cretaceous rock pore and fractured aquifer group; Yan’an formation (Jurassic System) rock pore and fractured aquifer group; Upper Triassic fractured aquifer group; Permian sandstone and Carboniferous thin limestone aquifer group.

Structurally, the overall structural complexity in this area is moderate. In general, the Hongliu Coal Mine takes on a linear structure in NW direction. The crisscrossed faults are widely distributed in the study area. According to statistics, 44 faults and five large-scale folds have been exposed by drilling in the study area.

The coal-measure strata in the mining area are in the Yan’an formation of medium Jurassic System. There are 18 coal seams. The main stable and minable coal seams are No. 2 and No. 4 with the average thickness of 4.61 m and 2.97 m, respectively.

### Division zones of the coal-roof strata after mining

**Figure 2:**
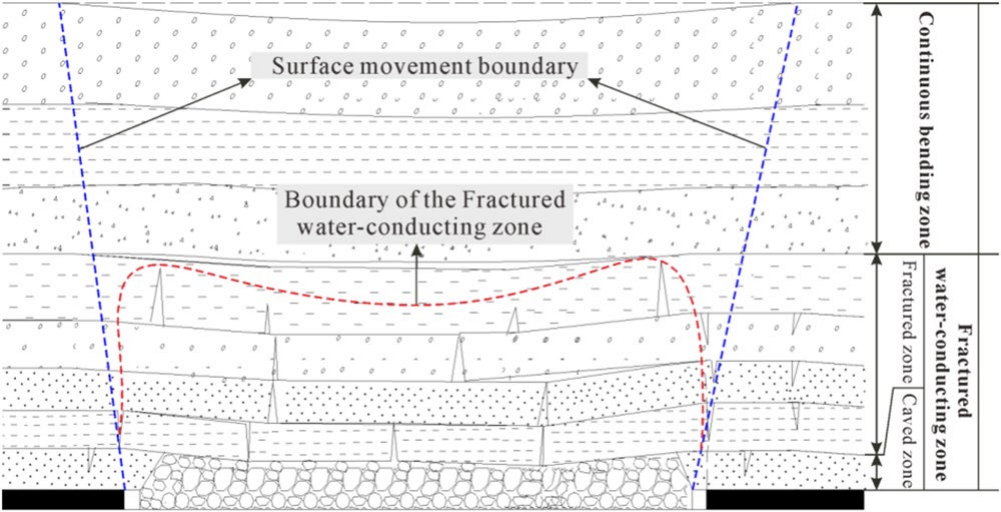
Division zones of the coal-roof strata after mining.

After the mining of the coal seam, the coal-roof strata are destroyed in various degrees, and have an obvious zoning property. According to the damage degrees and the movement characteristics, the coal-roof strata are divided into three zones: caved zone, fractured zone and continuous bending zone^4,17,32^, as illustrated in Fig. 2. The fractured water-conducting zone studied in this paper consists of the caved zone and fractured zone.

#### Caved zone

Caved zone is at the bottom of the overlying strata. With the moving forward of the mining working face, the immediate roof strata bear imbalance stress. When the load applied on the strata exceeding their bearing capacity, fractures generate. Finally, the strata crush, and the rocks irregularly fall into the void zone until it is filled. Thus, if an aquitard is located within the caved zone, its impermeability will become invalid in different degrees. So the caved zone provides ideal passages of the water from above aquifers to the working face.

#### Fractured zone

Fractured zone is above the caved zone, and the strata in this zone still maintain a certain continuity compared with the caved zone. The vertical fractures, inclined fractures and horizontal abscission-layer are heavily developed and distributed in the rocks at the bottom of this zone. The damage extent gradually decreases from the bottom of the fracture zone to the upper part, leading to the decrease of the fractures upward to the integrity rocks. This zone makes it possible that the fractures connect the aquifers, causing water inrushes from coal-roof strata. This zone is the main part of the water-conducting zone.

#### Continuous bending zone

Continuous bending zone refers to the strata between the fractured zone and the ground surface. The strata in this zone present the basic properties of downward movement without fractures developed within the rocks, especially the soft rock and loose soil strata. The movement of the strata almost hardly affects the impermeability of the aquitards in this zone, and it plays a protective role of the aquitards. A few fissures may appear in certain tension positions, but in general the strata maintain continuous^17^.

### Random forest regression (RFR)

RFR, introduced by Breiman in 2001, is an ensemble learning algorithm of multiple regression trees. Compared with simple decision trees, RFR runs efficiently on high-dimensional data sets, and it is more accurate and robust to noise^18,19^. Besides, RFR has great advantages over traditional intelligent algorithms^18–24^. On the one hand, it has a very fast learning process and can handle a large number of input variables while assessing the importance of variables. On the other hand, when building a forest, it can internally estimate the generalization error and estimating missing data can maintain high accuracy even if most of the data is lost.

**Figure 3:**
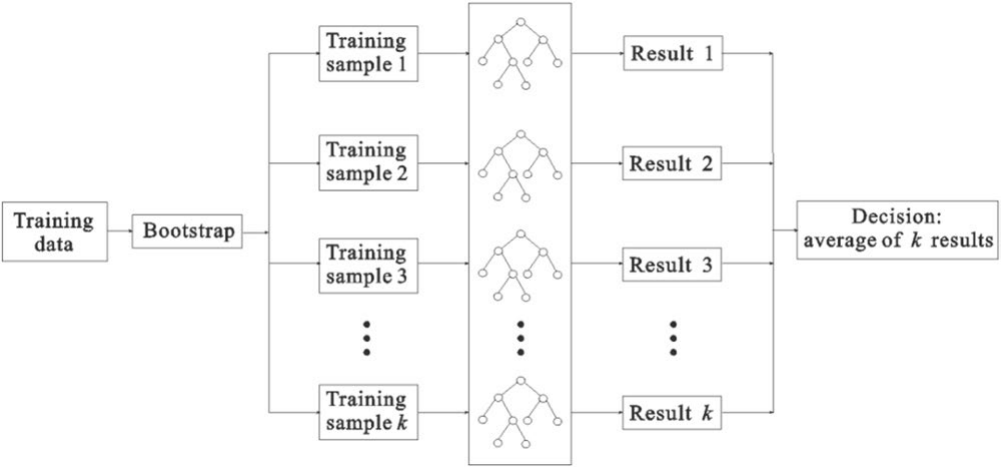
Sketch map of the RFR structure.

RFR is an ensemble of regression trees (RTs) to predict the value of a variable. It draws multiple samples based on the bootstrap resampling method from the original samples, and then constructs the decision trees model for the samples. Finally, the prediction output is obtained by calculating the average value of all prediction trees^18^. Figure 3 shows the sketch map of the RFR structure, and the specific implementation procedures of the RFR algorithm are as follows:

(1) Draw *k* samples randomly from the original training set *X* (*N* samples) using bootstrap resampling method, and then *k* regression trees are constructed. In this process, the probability that each sample wouldn’t be drawn is *p* = (1−1/*N*)^*N*^. If *N* tends to infinity, *p* ≈ 0.37, as indicates that about 37% of the samples in the original training set *X* are not drawn, these data are called out-of-bag (OOB) data. These OOB data can be used to be test samples.

(2) For *k* bootstrap samples, *k* unpruned regression trees are created respectively. In the tree growing process, for each node, *m* attributes are randomly selected from the total *M* attributes as internal nodes (*m* < *M*). Then, according to the minimum Gini index principle, an optimal attribute is selected from *m* attributes as a split variable to make the branches grow.

(3) The generated *k* regression trees constitute the final random forest regression model. The model estimation performance could be evaluated based on the indices: mean square error of OOB (*MSE*_*OOB*_) and coefficients of determination (${R}_{RF}^{2}$).

1 $$MS{E}_{OOB}=\frac{\sum _{i=1}^{n}{({y}_{i}-{\hat{y}}_{i})}^{2}}{n}$$

2 $${R}_{RF}^{2}=1-\frac{MS{E}_{OOB}}{{\hat{\sigma }}_{y}^{2}}$$

where *n* is the total number of the OOB samples; *y*_*i*_ is the observed output value; *ŷ*_*i*_ is the predicted output obtained by the generated RFR regression model; ${\hat{\sigma }}_{y}^{2}$ is the predicted variance of the OOB output.

### Variables importance measures

The RFR model provides two ways to calculate the importance degree of each variable index: mean decrease in Gini index and mean decrease in accuracy^18–20^.

The mean decrease in Gini index means the total impurity decrease of each variable at each tree node. The method evaluates the importance of the variables by calculating the Gini index based on the Equation (3), and then accumulates the total impurity decrease of all the trees.

3 $${I}_{Gini}=1-\sum _{i=1}^{N}{p}_{i}^{2}$$

where *p*_*i*_ is the probability of the samples belonging to the *i*-th leaf; *N* is the number of the leave; *I*_*Gini*_ is the Gini index.

The basic principle of the OOB error estimation method is: when the noise is added to a related feature which plays an important role in the accuracy, the prediction accuracy of the RFR will decrease significantly. The main procedures are as follows: firstly, for the generated RFR, the OOB error *e*_*t*_ of each decision tree is calculated according to the OOB data; secondly, the *j*-th eigenvalue *X*^*j*^ of the OOB data is changed randomly (namely the noise interference is added artificially); then, the OOB data with noise are used to test the performance of the RFR and a new OOB error ${e}_{t}^{j}$ is obtained. Finally, the importance degree of the variable *X*^*j*^ can be calculated according to the Equation (4):

4 $$I({X}^{j})=\frac{1}{n}\sum _{t=1}^{n}({e}_{t}^{j}-{e}_{t})$$

where *X*^*j*^ is the *j*-th eigenvalue of the OOB data; *e*_*t*_ is the initial OOB error; ${e}_{t}^{j}$ is the OOB error with noise; *n* is the number of the decision trees; *I*(*X*^*j*^) is the importance of the variable *X*^*j*^. The greater the OOB error caused by the change of the variable *X*^*j*^, the more the decrease in accuracy, indicating the more important the variable is.

### Construction of the main controlling factors system

The development of FWCZ of coal-roof strata is influenced by multiple factors. And it has a complex nonlinear relationship with the strata geological features, rock mechanics and mining conditions^17^. Based on a large number of field observations for fully-mechanized mining and theoretical studies, five main controlling factors were selected, including mining depth, mining height, lithology type of the overlying strata, working-face length and coal-seam dip angle. A brief overview of the five factors is described as follows.

#### Mining depth

According to the theories of mining engineering geology and rock mechanics, the situ stress of the strata around the underground excavation space has a great impact on the destruction scope of the surrounding rock. Generally, the primary rock stress of the surrounding rock is proportional to the mining depth. With the increase of the mining depth of the coal seam, the *in situ* stresses and the displacement of the overlying rock gradually increase, which will lead to more fractures developed in the coal-roof strata.

#### Mining height

Mining height is the decisive factor of the fractured zone height. The greater the mining height, the larger the range of the coal-roof plastic zone. And a greater space available to the caving rock will form, resulting in a greater height of the fractured zone. In the traditional empirical formula prediction method, mining height is the only factor that controls the FWCZ height.

#### Lithology type

When the overlying rock is disturbed by the mining activities, the brittle rock with higher hardness (such as limestone and sandstone) is apt to crack and produce fractures. While, for the soft rock (such as mudstone and shale), the plastic deformation mainly occurs, and fractures rarely appear. After the extraction of the coal seams, the compressive strength of the overlying rock directly affects the rock failure degree. The rock with a greater compressive strength will be not prone to be destroyed. Generally, according to the uniaxial compressive strength of the rock, the lithology of the overlying strata is classified into four types^13–15^: hard, medium hard, medium soft and soft, with the quantitative values of 4, 3, 2 and 1, respectively.

#### Working-face length

Working-face length, like the mining height, is an index that reflects the influence of the mining space size on the fractured water-conducting zone. According to the material mechanics theory, the curvature of a rock beam with two ends fixed is proportional to the span. The greater the length of the working face, the greater the downward curvature of the coal-roof strata. Thus, the break probability of the rock beam increases, resulting in a higher height of the fractured zone.

#### Coal-seam dip angle

The influence that the coal-seam dip angle on the overlying strata is mainly embodied in the different failure forms of the strata. When the coal seam is horizontal, the form of the fractured zone is nearly symmetrical, showing a saddle shape. With the increase of the dip angle, the failure form of overlying rock gradually develops into parabola and arch shapes.

## Results and Discussion

### Datasets used

**Figure 4:**
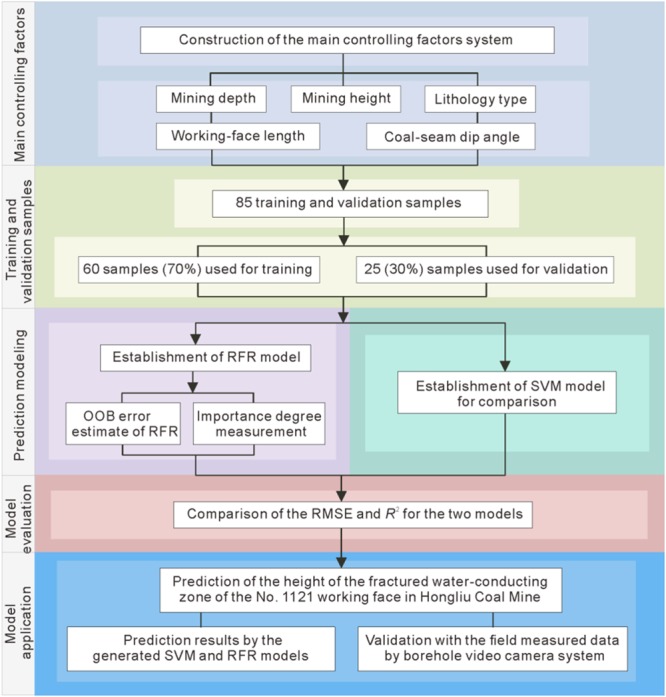
Detailed flowchart of the proposed methodology.

The collection of the datasets is the most important part for any machine learning algorithm. In this study, 85 field measured datasets for fully-mechanized mining were collected from several large-scale coalmines in North China, referring to the previous research documents^13–17^. Each case contains the field measured data of the aforementioned five main-controlling factors and the height of FWCZ. Of the 85 datasets, 60 (70%) were randomly selected for training (Table 1), while the remaining 25 (30%) for model testing. Figure 4 shows the detailed flowchart of the methodology used in this study.

**Table 1:** Field measured sample datasets for model training.

No.	Sample source	Mining depth (m)	Mining height (m)	Lithology type	Working-face length (m)	Coal-seam dip angle (°)	Height of fractured water-conducting zone (m)
1	No. 3_2_41 working face in Qidong Coal Mine	550	2.4	4	180	15	55.32
2	No. 8 coal seam in Yangzhuang Coal Mine	320	1.7	4	65	6	27.5
3	No. 2 coal seam in Tiebei Coal Mine	125	3	1	150	5	22
4	No. 4320 working face in Xinglongzhuang Coal Mine	450	8	4	170	8	86.8
5	No. 4308 working face in Dongtan Coal Mine	43	3	4	30	60	35
6	No. 16 coal seam in Zhaopo Coal Mine	120	1.2	2	75	8	31
7	No. 3 coal seam in Wulanmulun Coal Mine	101.1	2.2	3	158	1	63
8	No. 13013 coal seam in Baodian Coal Mine	417	2.9	4	80	4	68
9	No. 2 coal seam in Guantai Coal Mine	300	4	2	75	2	120
10	No. 8 coal seam in Luling Coal Mine	276	4.5	1	350	7	17.2
11	No. 7 coal seam in Fangezhuang Coal Mine	84	4	2	108	3	30
12	No. 1203 working face in Daliuta Coal Mine	49	4	1	135	5	45
13	No. C13-1 working face in Panxie Coal Mine	117	3.4	2	205	2	72
14	No. 1 coal seam in Xinji Coal Mine	290	6	1	645	8	85.6
15	No. 4 coal seam in Liuhualing Coal Mine	89	2.03	4	69	7	45.86
16	No. 16 coal seam in Laoshidan Coal Mine	200	1.5	1	45	0	4.5
17	No. 2 coal seam in Kongji Coal Mine	200	8	4	89	76	48
18	No. 11 coal seam in Tongting Coal Mine	230	2	1	85	37	52.5
19	No. 1 coal seam in Qilianta Coal Mine	56	4.3	4	55	0	42.5
20	No. 3_1_107 working face in Luxi Coal Mine	350	2.5	2	135	5	20
21	No. 9 coal seam in Luling Coal Mine	284	7	2	130	3.5	26
22	No. 7141 working face in Qidong Coal Mine	520	2.3	3	174	12	50.675
23	No. 3241 working face in Qidong Coal Mine	509	2.25	3	180	12.5	34.925
24	No. 7130 working face in Qidong Coal Mine	402.5	3	3	170	12	19.6
25	No. 1013 working face in Wugou Coal Mine	386.5	3.1	3	150	10	40.79
26	No. 1017 working face in Wugou Coal Mine	380	3.5	3	180	6	45.84
27	No. 345 working face in Qinan Coal Mine	395.5	3.45	3	160	14	26.7
28	No. 1031 working face in Taoyuan Coal Mine	384.2	2.65	3	190.5	21	33
29	No. 1062 working face in Taoyuan Coal Mine	306	3	3	150	28	33.615
30	No. 745 working face in Haizi Coal Mine	404.5	2.3	3	95	18	19.5
31	No. 1031 working face in Haizi Coal Mine	313.5	2.4	3	65	6	21.9
32	No. 841 working face in Zhuxianzhuang Coal Mine	342.5	3.8	3	114	13	28.455
33	No. 821 working face in Zhuxianzhuang Coal Mine	338.5	1.9	3	115.5	20	20.995
34	No. 822 working face in Zhuxianzhuang Coal Mine	316	1.9	3	165	12	26.085
35	No. 721 working face in Zhuxianzhuang Coal Mine	296	1.9	4	95.5	15	17.84
36	No. II 865 working face in Zhuxianzhuang Coal Mine	493.75	13.43	3	130	15	93.175
37	No. 8212 working face in Xutong Coal Mine	395	2.5	3	178	9	26.33
38	No. 7126 working face in Xutong Coal Mine	478.5	2.5	3	180	8	33.755
39	No. 16028 working face in Paner Coal Mine	340	1.8	2	178	3	19.69
40	No. 1207 working face in Paner Coal Mine	319	2	2	148	5	17.155
41	No. 1201(3) working face in Paner Coal Mine	311	2	2	85	3	19.11
42	No. 1201(1) working face in Paner Coal Mine	327.5	2	2	78	7	22.995
43	No. 12128 working face in Paner Coal Mine	355.5	2	2	125	3	23.865
44	No. 12118 working face in Paner Coal Mine	349	2	3	130	5.5	22.31
45	No. 12117 working face in Paner Coal Mine	363	2	3	180	8	16.845
46	No. 1701(3) working face in Pansan Coal Mine	447	2	3	107	4	30.965
47	No. 1711(3) working face in Pansan Coal Mine	420.5	2.8	3	135	3	41.13
48	No. 1211(3) working face in Pansan Coal Mine	509.5	3	2	140	10	26.01
49	No. 14032(3) working face in Panyi Coal Mine	383	2.2	2	125	5	13.035
50	No. 14021(3) working face in Panyi Coal Mine	376.5	2	2	124	5	12.675
51	No. 1401(3) working face in Panyi Coal Mine	391	1.8	2	125	5	14.29
52	No. 1402(3) working face in Panyi Coal Mine	404	2.2	2	150	6	21.195
53	No. 1412(3) working face in Panyi Coal Mine	415	3.4	3	120	8	30.085
54	No. 1121(1) working face in Panyi Coal Mine	418	1.8	3	120	6	24.69
55	No. 2622(3) working face in Panyi Coal Mine	552.5	5.8	3	182	8	44.36
56	No. 1121(3) working face in Panyi Coal Mine	490.5	6	3	182	7	44.19
57	No. 1211(3) working face in Xieqiao Coal Mine	445	4	3	198	8	29.265
58	No. 1221(3) working face in Xieqiao Coal Mine	490.5	5	3	172	8	52.76
59	No. 1221(3) working face in Zhangji Coal Mine	605.5	3	3	136	2	38.185
60	No. 1212(3) working face in Zhangji Coal Mine	516	3.9	3	205	2	31.765

### Establishment of the RFR model

**Figure 5:**
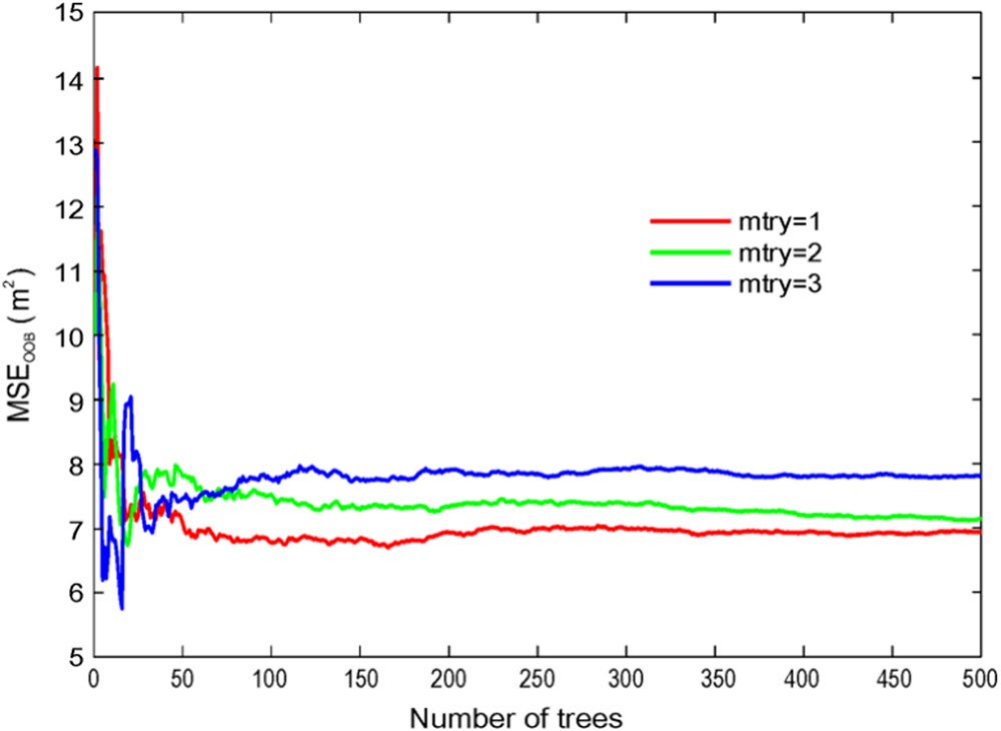
The OOB error of the RFR model.

In the RFR, two parameters are required to define: the number of trees in the forest (*ntree*), and the number of the random variables of the split nodes (*mtry*). To maximize the model accuracy, it is necessary to optimize the combination of the parameters *mtry* and *ntree*^18^. When *ntree* is defined with a small value, the RFR prediction error is uncontrollable and the model performance cannot achieve the optimal identification. Conversely, if the parameter *ntree* is too large, the computation time and required memory will increase accordingly. By repeated operation, it is found that when *ntree* = 200, the MSE_OOB_ tends to be stable and the model does not tend to over fitting. According to Breiman^18^, *mtry* &lt; *M*. In this case study, there are five variables, namely *M* = 5. To assess the optimal value of *mtry*, three RFR models were created for *mtry* = 1, *mtry* = 2 and *mtry* = 3 (Fig. 5). Figure 5 shows the change of the error depending on the number of the trees *ntree*. The results show that when *ntree* = 200, the error of the model is stable, and when *mtry* = 1, the MSE_OOB_ is lowest at about 6.9 m^2^. Therefore, considering both the accuracy and computation cost, the two optimized parameters of the RFR are as follows: *mtry* = 1 and *ntree* = 200.

**Figure 6:**
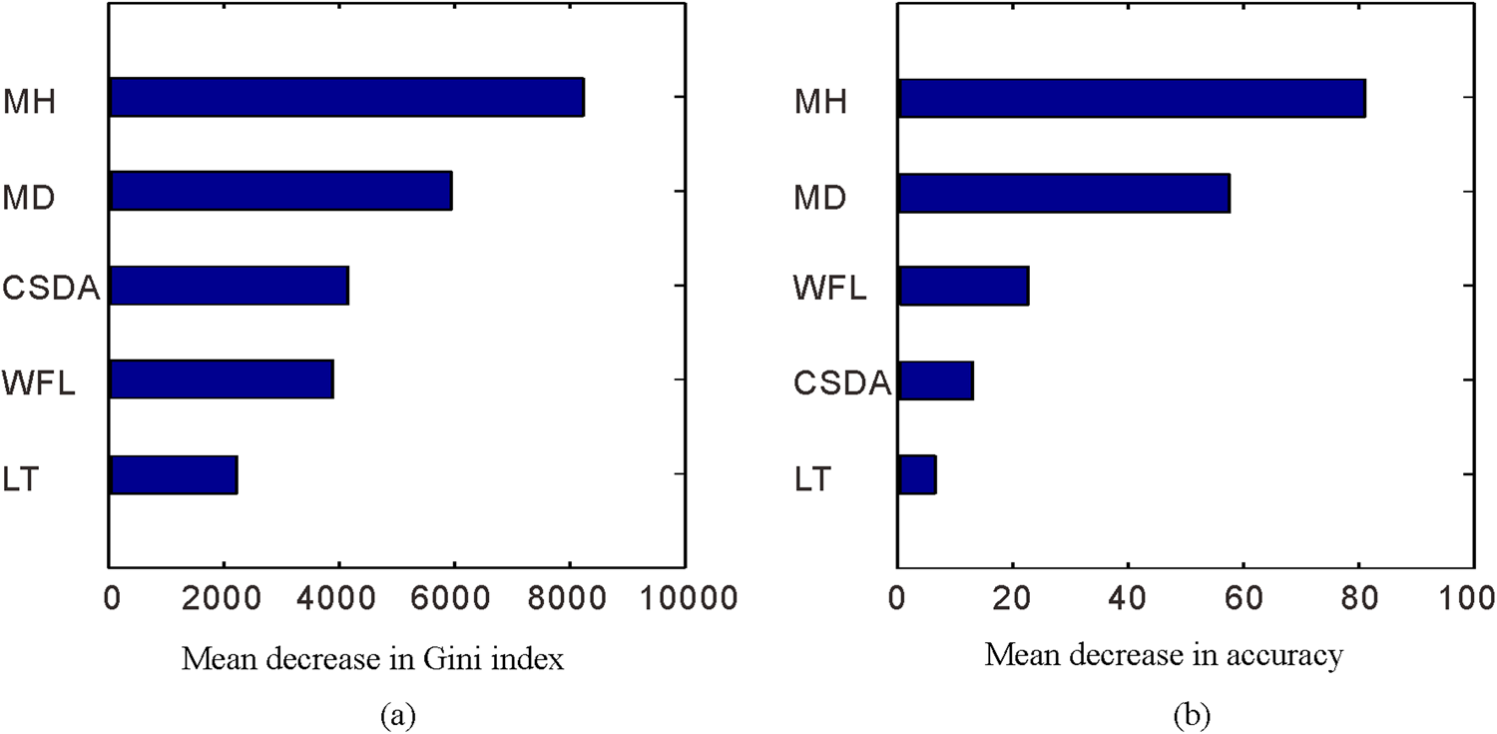
Importance degree of the main controlling factors determined by two ways: (**a**) Mean decrease in Gini index; (**b**) Mean decrease in accuracy. (MH: mining height; MD: mining depth; CSDA: coal-seam dip angle; WFL: working-face length; LT: lithology type).

The contribution of each factor to the generated RFR model is shown in Fig. 6. As shown, the importance degree of each factor is measured based on two ways: mean decrease in Gini index and OOB mean decrease in accuracy. According to the Gini index, mining height and mining depth have the highest importance, followed by coal-seam dip angle and working-face length, while lithology type has the lowest importance. Regarding the OOB mean decrease in accuracy, the order of the importance degree is consistent with the result obtained by Gini index method. Based on both of the features of importance, mining height and mining depth are the two most important factors out of the five factors, as suggests that they contribute overwhelmingly to the development of the FWCZ height.

### SVM model for comparison

For comparison, support vector machine (SVM) regression model was also used for the prediction of the height of fractured water-conducting zone. SVM has superior prediction performance in various fields for data modeling and function optimization because of its ability to represent non-linearities^23^. The radial basis function (RBF) was adopted as the kernel function, and the two main parameters RBF kernel coefficient *γ* and penalty coefficient *C* were determined as 0.1 and 0.5. And then the SVM regression model was constructed using the same training data aforementioned.

### Model evaluation

The model evaluation is an important procedure before the model application. The root mean square error (RMSE) and the coefficient of determination *R*^2^ were utilized to evaluate the performance of the two generated regression models. RMSE is generally used for measuring the residual errors, and it reflects the difference between original and modeled values. The lower the RMSE, the better the model performs. *R*^2^ provides a measure of how well the predicted output of the regression model fits the observed data. The value of *R*^2^ varies between 0 and 1. A higher *R*^2^ indicates that the regression model fits the data better. The two indices are defined as follows:

5 $${\rm{RMSE}}=\sqrt{\frac{1}{n}\sum _{i=1}^{n}{({y}_{i}-{\hat{y}}_{i})}^{2}}$$

6 $${R}^{2}=1-\frac{{\sum }_{i=1}^{n}{({y}_{i}-{\hat{y}}_{i})}^{2}}{{\sum }_{i=1}^{n}{({y}_{i}-\bar{y})}^{2}}$$

where *n* is the total number of the test samples; *y*_*i*_ is the observed output value of the test samples; *y*_*i*_ is the predicted value by the generated models; *ŷ*_*i*_ is the average output value of the test samples.

**Figure 7:**
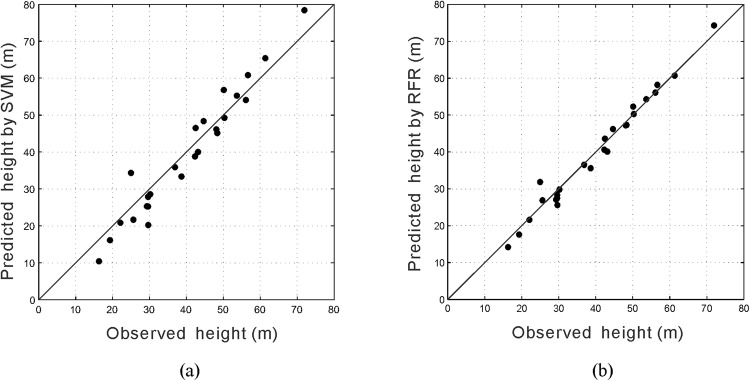
Comparison of the observed and predicted height with the test data by using: (**a**) SVM; (**b**) RFR.

Table 2 lists 25 sample datasets for model testing to evaluate the performance of the models. Using the two regression models generated above, the heights of FWCZ of the 25 cases were predicted. Figure 7 shows the predicted value against the observed data with the test data using SVM and RFR, respectively. Based on the Equations (5) and (6), the RMSE and *R*^2^ of the two models were calculated as Table 3. As it shown, the RFR model has the lower RMSE and higher *R*^2^ with the value of 2.363 and 0.968, respectively (compared to 4.396 and 0.902 for SVM model). Therefore, it is concluded that both models are reasonable, and RFR has a better performance compared with the SVM.

**Table 2:** Field measured sample datasets for model testing.

No.	Sample source	Mining depth (m)	Mining height (m)	Lithology type	Working-face length (m)	Coal-seam dip angle (°)	Height of fractured water-conducting zone (m)
1	No. 1215(3) working face in Zhangji Coal Mine	520.5	3	3	202	2	33.365
2	No. 1242(1) working face in Gubei Coal Mine	620	3.1	4	240	3.5	20.215
3	No. 7_1_92 working face in Kongzhuang Coal Mine	220	5.3	3	120	25	46.5
4	No. S4101 working face in Pingshuo Coal Mine	360	7.69	3	220	3	45.125
5	No. ZF2801 working face in Xiagou Coal Mine	347	9.9	3	100	2	79.255
6	No. 5306 working face in Xinglongzhuang Coal Mine	412	6.9	3	160	4	38.8
7	No. 6206 working face in Wangzhuang Coal Mine	316	5.9	3	248	4.5	60.81
8	No. I03(2) working face in Laogongyingzi Coal Mine	240	3.5	1	195	7	21.675
9	No. I03(4) working face in Laogongyingzi Coal Mine	240	3.5	1	195	7	17.445
10	No. 3202 working face in Wangpo Coal Mine	474.16	5.8	3	230	4	65.395
11	No. 93_1_01 working face in Nantun Coal Mine	541.5	5.28	3	175	6.5	49.25
12	No. 1301 working face in Jisan Coal Mine	480	6.3	3	170	4	46.13
13	No. 1305 working face in Dongtan Coal Mine	600	8.78	3	223.35	6	54.08
14	No. 2308 working face in Xinglongzhuang Coal Mine	332.85	7.15	3	160	5	16.115
15	No. 2306 working face in Xinglongzhuang Coal Mine	319.2	8.2	3	160	7.5	27.84
16	No. 2302 working face in Xinglongzhuang Coal Mine	278.15	8.7	3	170	8	28.56
17	No. 2300 working face in Xinglongzhuang Coal Mine	282	8.55	3	140	5	25.255
18	No. 23S2 working face in Xinglongzhuang Coal Mine	258.55	8.45	3	175	3	20.85
19	No. 2303 working face in Xinglongzhuang Coal Mine	286.45	7.8	3	150	8	35.9
20	No. 1314 working face in Baodian Coal Mine	350	8.5	3	169	6.5	55.255
21	No. 2605 working face in Yangcun Coal Mine	187.5	1.2	3	300	10	10.41
22	No. 63_1_10 working face in Nantun Coal Mine	368.05	5.77	3	125	6	48.35
23	No. 2186 working face in Donghuantuo Coal Mine	420	3.4	3	70	23	56.8
24	Fangezhuang Coal Mine	173	1.9	4	70	20	25.3
25	No. 1672 working face in Qianjiaying Coal Mine	446	3.8	4	143	17	40

**Table 3:** RMSE and *R*^2^ of the RFR and SVM models.

Prediction model	RMSE (m)	*R* ^2^
SVM	4.396	0.902
RFR	2.636	0.968

### Model application

#### Engineering background and predicted results

The No. 1121 working face of the No. 2 coal seam, located in the center of Hongliu Coal Mine, is the initial mining face of the mine. The length of the working face is 1379 m, and the average mining depth is 265 m. The fully-mechanized longwall mining method is adopted in the mining process. The No. 2 coal seam belongs to the Yan’an Formation of the Jurassic System, with an average thickness of 5.28 m. The dip angle of the No. 2 coal seam varies from 5° to 15°, with the average value of 10°.

**Figure 8:**
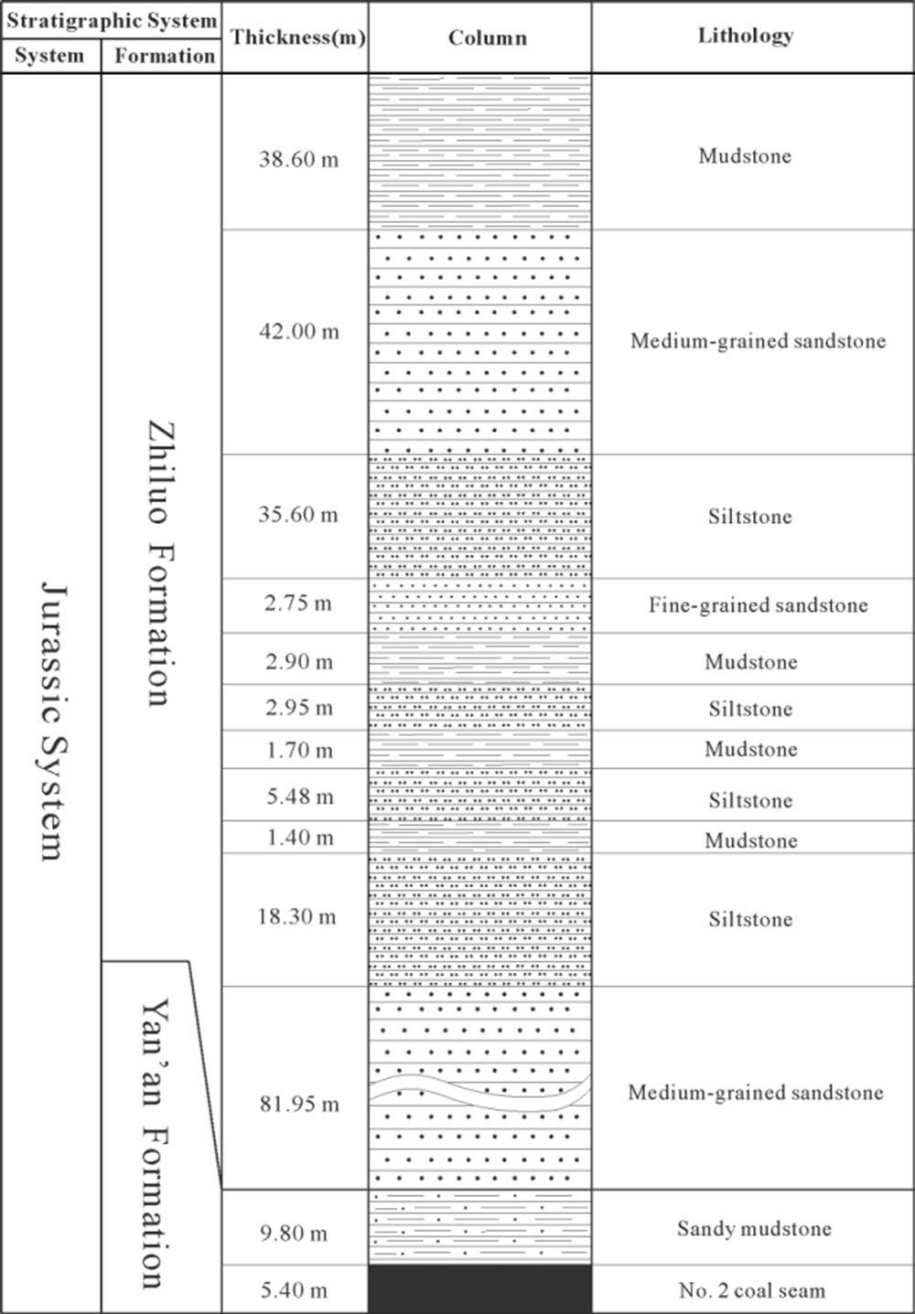
Typical geological column of the No. 1121 working face overlying strata.

Figure 8 displays the typical geological column of the No. 1121 working face overlying strata. As it shown, the strata directly overlying the coal mainly consist of the silt and fine sandstones in the lower Zhiluo formation, which are considered as aquitards. The average total thickness of these strata is 52.2 m. According to the rock division rule aforementioned, the sandstone is considered to be hard rock, so the lithology type of the strata is quantified as 4. The first aquifer overlying the No. 2 coal seam is about 51.28 m distance from the coal. It consists of grit sandstones with great thickness, and it has a rich water-abundance property. Thus, in order to evaluate the risk of water inrushes from overlying the coal seam and take corresponding measures, it is necessary to precisely predict the height of the FWCZ. By applying the above generated SVM and RFR models to the No. 1121 working face of the No. 2 coal seam, the height of FWCZ is predicted as 64.17 m and 62.96 m, respectively.

#### Practical situation of the No. 1121 working face

When the No. 1121 working face moved forward about 56 m, a large amount of water from the overlying aquifer leaked into the working face. The maximum water inflow was up to 1817 m^3^/h, so the mining operation had to be terminated. For drawing up the water-inrush prevention measures scientifically, the borehole video camera system (BVCS) was used to observe the height of the fractured water-conducting zone. BVCS is an exploration technology which can directly observe the inner conditions of the boreholes based on the optics theory. The system can be used to observe the strata lithology, geological structures properties, fracture-zone development conditions, groundwater levels change and so on^10^.

**Figure 9:**
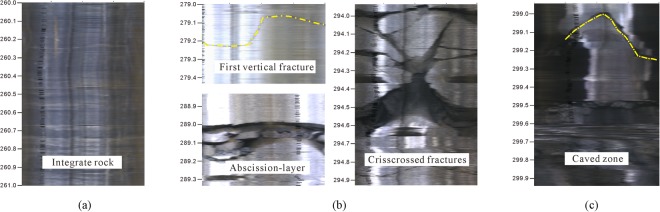
Video camera images of the borehole HL-1: (**a**) Integrate rock without fracture; (**b**) Fractured zone with various forms of fractures; (**c**) Caved zone.

In this study, the BVCS was used to observe the change of the fractures development degree with the increase of the borehole depth and determine the top boundaries of the fractured zone and the caved zone. Figure 9 shows the video camera images of the borehole HL-1. According to the images, the rocks above 279.07 m are sandstone and mudstone interbed, and they are relatively integrated except that a few small cracks in the horizontal direction appear in certain local positions (Fig. 9a).

Figure 9b shows the fractured zone images with various forms of fractures: a nearly vertical fracture with a small width appears at the borehole depth of 279.07–279.27 m; there are many abscission-layer phenomena between 286.3 m and 294.68 m; the crisscrossed fractures with large displacements are distributed in the rocks below 294 m. Therefore, according to the fractures development conditions described above, the depth of 279.07 m is considered as the top boundary of the fractured zone.

As Fig. 9c shown, the rocks below the depth of 298.9 m were damaged seriously, and there is a vast void area with obvious mining collapse characteristics. So the depth of 298.9 m is determined as the top boundary of the caved zone.

Based on the formula proposed by China Coal Industry Bureau^20^, the height of FWCZ of coal-roof strata can be calculated as follows:

7 $$H=H^{\prime} -h-M$$

where *H* is the maximum height of FWCZ (m); *H*′ is the depth of the coal-seam floor (m); *h* is the depth of the fractured zone’s top boundary (m); *M* is the thickness of the mining coal seam (m).

According to the drilling data and the video camera images of borehole HL-1, the depth of the No. 2 coal-floor is 345.88 m, and the thickness of the coal seam is 5.28 m. Therefore, combined with the Equation (7), the height of FWCZ is calculated to be *H* = (345.88–279.07–5.28) = 61.53 m. Table 4 shows the comparison between the field measured data and the prediction results obtained by the proposed methods.

**Table 4:** Comparison between the field measured data and the prediction results obtained by the SVM and RFR.

Model	Prediction results (m)	Field measured data by BVCS (m)	Absolute error (m)	Relative error (%)
SVM	64.17	61.53	2.64	4.29
RFR	62.96		1.43	2.32

The results show that compared with the field measured data, the SVM and RFR methods have the relative error of 4.29% and 2.32%, respectively. It indicates that both of the prediction results are generally in good agreement with the field-observed result, and the RFR model has a better performance in the application of the study area, which is in accordance with the above conclusion.

## Summary and Conclusions

To ensure the safe production of coal mines, this study proposed a prediction model of the height of FWCZ based on RFR. RFR is a robust machine learning method that can be used to evaluate the variable importance and predict the height of FWCZ. Compared with the traditional MLAs, RFR has numerous advantages, especially, its high prediction accuracy and it is well suitable for the problems with unclear priori knowledge and incomplete data. For the objective problems faced in this study, for instance, the lack of data samples, the RFR model can still maintain a high degree of accuracy. Then, the RFR model was applied to Hongliu Coal Mine in Northwest China. And the main conclusions are reached as follows.

1. Five variables were selected to construct the main controlling factors system. And according to the importance degree measurement by the mean decrease in Gini index and OOB mean decrease in accuracy, mining height and mining depth are the top two most important factors out of the five variables.
2. For comparison with the generated RFR model, a SVM model was also constructed using the same training datasets. By the validation of the two models, the RFR model has the lower RMSE and higher *R*^2^ with the value of 2.363 and 0.968, respectively (compared to 4.396 and 0.902 for SVM model).
3. The two generated models were applied to the No. 1121 working face in Hongliu coal mine to verify the effectiveness of the models. The prediction heights of the FWCZ by using RFR and SVM are 62.96 m and 64.17 m, respectively. Field measured data by borehole video camera system is 61.53 m, and the RFR and SVM have the relatively error of 2.32% and 4.29%, respectively. It is concluded that RFR has a better performance in the application of the study area compared with the SVM.
4. This study shows the potential to provide a novel approach to predict the height of FWCZ. The results provide a reference for water-inrush risk management, prevention and reduction in the study area.
